# The effect of anthropogenic drivers on spatial patterns of mangrove land use on the Amazon coast

**DOI:** 10.1371/journal.pone.0217754

**Published:** 2019-06-26

**Authors:** Sanae N. Hayashi, Pedro Walfir M. Souza-Filho, Wilson R. Nascimento, Marcus E. B. Fernandes

**Affiliations:** 1 Laboratório de Ecologia de Manguezal, Instituto de Estudos Costeiros, Campus Bragança, Universidade Federal do Pará, Bragança, Pará, Brazil; 2 Laboratório de Geoprocessamento e Educação Financeira e Ambiental, Campus Capanema, Universidade Federal Rural da Amazônia, Capanema, Pará, Brazil; 3 Laboratório de Análises de Imagens do Trópico Úmido, Instituto de Geociências, Universidade Federal do Pará, Belém, Pará, Brazil; 4 Instituto Tecnológico Vale, Belém, Pará, Brazil; Imperial College London, UNITED KINGDOM

## Abstract

Mangroves play an essential ecological role in the maintenance of the coastal zone and are extremely important for the socioeconomics of coastal communities. However, mangrove ecosystems are impacted by a range of anthropogenic pressures, and the loss of this habitat can be attributed primarily to the human occupation of the coastal zone. In the present study, we analyzed the spatial patterns of land use in the mangrove of the Brazilian Amazon coast, and evaluated the anthropogenic drivers of this impact, using a remote sensing approach. We mapped the road network using RapidEye images, and human settlements using global data. The results of these analyses indicate that the Brazilian Amazon coast has a low population density and low rates of anthropogenic impact in most of the coastal microregions investigated, factors that contribute to the maintenance and conservation of the region’s mangrove. The study also revealed that the paved road network is one of the principal drivers of land use in the mangrove, whereas other factors, such as population density, urban centers, and the number of settlements are much less important. While the region has 2024 km of paved highways, unpaved roads (17,496 km) facilitate access to the mangrove, with approximately 90% of anthropogenic impact being recorded within a 3 km radius of these roads. While the network of paved highways is relatively reduced in extension, preventive measures are urgently required to impede any major shift in the current scenario, caused by the expansion of major development programs. The results of the study indicate that biophysical, economic, and political factors may also contribute to the reduction, stability, and development of one of the world’s largest areas of mangrove forest.

## Introduction

Mangroves play a fundamental ecological role in the maintenance of the coastal zone and have enormous socioeconomic importance for traditional local communities [1–4]. Mangroves offer many ecosystem services [5], such as the protection of the coastline from catastrophic and erosive events [6,7], conserving and recycling nutrients [8], and water regulation [9], in addition to providing shelter, refuge, and feeding resources for local animals [10]. Mangroves also play a key role in human sustainability and livelihoods. The biodiversity of these forests is exploited by the human populations of tropical coastal-estuarine regions for their subsistence needs, including the harvesting of food and fuel wood, and the extraction of lumber for construction [11, 12]. The mangrove ecosystem is also among the world’s most dynamic and productive coastal environments [13], with a primary production equivalent to that of the tropical rainforest [14]. One other service provided by the mangrove is its role as a carbon sink. This ecosystem is also among the tropical ecosystems that are the richest in carbon anywhere in the world [15]. More than half of all mangrove carbon stocks are found in Indonesia, Brazil, and Papua New Guinea [16]. Thus, mangrove forests have enormous potential for the marketing of carbon credits for the reduction of the emission of greenhouse gases [17], reinforcing conservation strategies and contributing to the mitigation of climate change [16,18].

Despite their value, mangroves suffer increasing pressure from expanding human activities [19–21], and are being reduced at a rate of 1–2% per annum, worldwide [22–25]. In Brazil, while all mangrove habitat is considered to be an Area of Permanent Preservation (APP) [26], an area of 500 km^2^ has been lost over the past 25 years, primarily on the country’s southern coast [18]. Worldwide, mangroves are being lost to make way for farmland, aquaculture operations, and urban development, mining, and logging, including clear-cutting [27–35]. The salt flat zone, which is an important feature of the mangrove ecosystem [36, 37], is also pressured by economic activities, in particular shrimp farming and the production of sea salt [5, 29–35]. In Brazil, this scenario is accentuated by the fact that salt flat is not considered to be a part of the mangrove ecosystem, and is thus not legally protected as an APP. This has led to approximately 10% of the country’s salt flats being impacted for the production of salt and shrimp [38, 39]. All these activities impact the mangrove. Shrimp farming, for example, is one of the principal threats, through the degradation of the mangrove [40,41], as are deforestation and the over-exploitation of fishery resources [42]. The extraction of timber, even on a small scale, is one other activity that contributes to the degradation of the mangrove. The recuperation of impacted mangrove on hyper-saline soils can be a slow process, which may make this activity unsustainable [43]. On a larger scale, the extraction of mangrove ecosystems can alter the characteristics of the soil, and reduce the abundance and diversity of plants [44]. As a consequence of human pressures, then, the ongoing loss of mangroves may result in a reduction of the ecosystem services provided by these environments. This will entail serious ecological and socio-environmental impacts for the traditional coastal communities that depend on the mangrove for their subsistence [45].

Around 10% of the world’s population lives in coastal regions [46], which have a higher population density than the global mean [47]. Brazil is typical of this scenario, and in fact, 26% of the country’s population inhabit coastal urban centers, which represent only 1% of its total area [48]. Global estimates indicate that, by 2015, approximately 120 million people lived in areas of mangrove, and this population is highly dependent on the resources of this ecosystem for its survival [49]. The worldwide loss of mangroves is due primarily to the growth and development of human populations in coastal zones [35]. Other factors also influence this process, such as the concentration of urban areas [35], the extension of roads [27, 50, 51], and infrastructure and proximity to major cities and towns [52].

Given the importance of the mangrove and the seriousness of the threats faced by this ecosystem, the mapping of land cover and use is one of the most important initial steps toward the development of measures to combat and prevent degradation, and support habitat restoration. It is fundamentally necessary to advance these measures, given that multiple anthropogenic pressures may influence the loss of mangroves, which vary considerably in their characteristics and dynamics in different regional and local contexts. To better comprehend the dimensions of the human pressures on the mangrove, it is necessary to analyze how these drivers interact and stimulate the use of this ecosystem, considering the specific features of each region. In this context, the application of remote sensing and Geographic Information Systems (GISs) are essential tools for the effective detection of intrinsic human impacts on the mangrove [52–55]. These tools can also provide important data on demographic processes [56, 57] and the infrastructure necessary for regional development [58–61]. This interdisciplinary approach is essential for the development of effective measures and strategies to mitigate negative impacts and guarantee the conservation of the mangrove.

The present study analyzes spatial patterns and presents evidence of the effects of anthropogenic drivers on the use of land in the mangrove of the Brazilian Amazon coast. RapidEye images were used to determine the region’s road network. This analysis was complemented with the evaluation of population density and the patterns of human occupation in this coastal region. This unique multidisciplinary approach was used to determine the anthropogenic factors that drive mangrove land use, and the long-term implications of this process for the world’s largest continuous tract of mangrove forest.

### Study area

**Fig 1:**
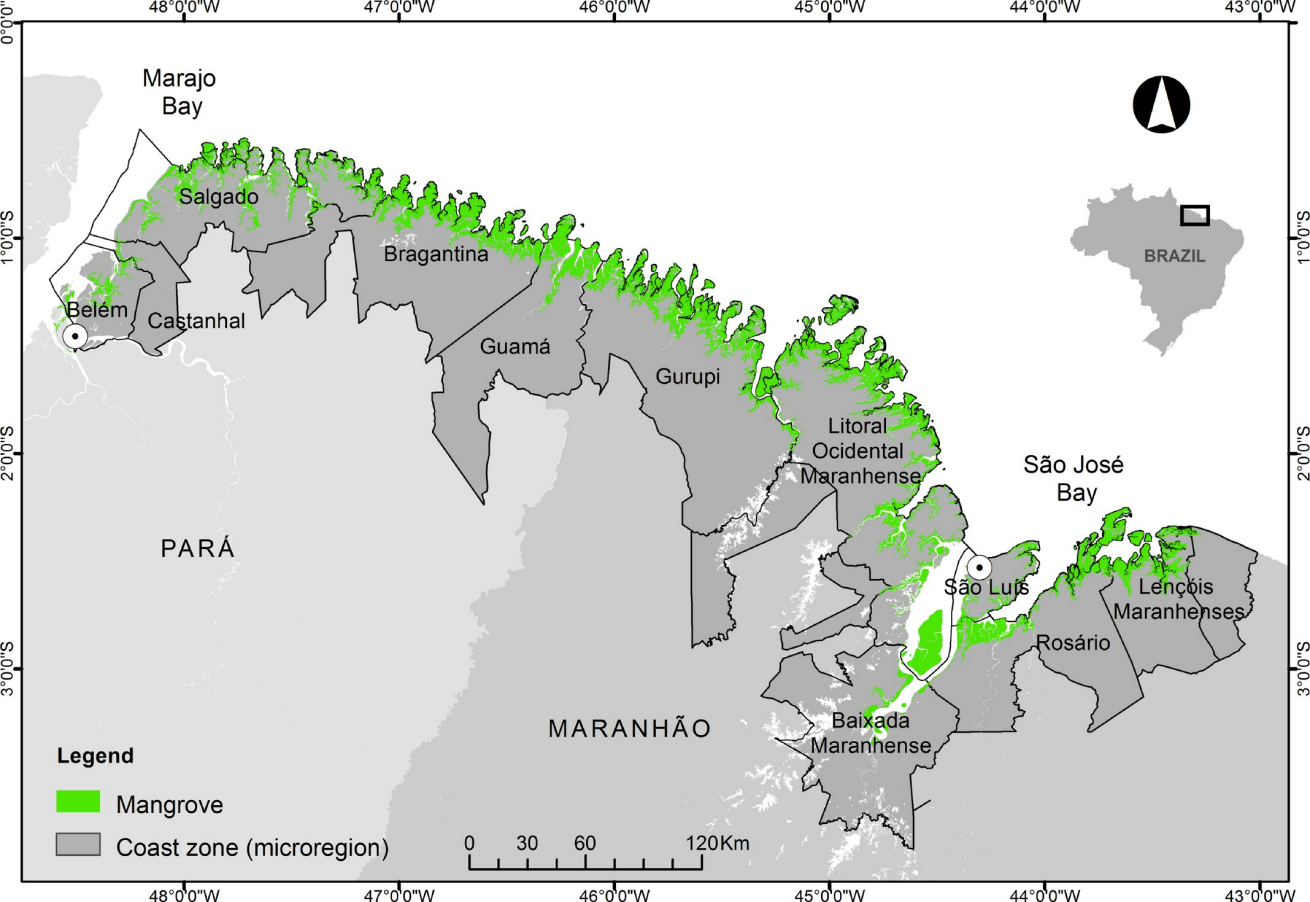
Map of the study area. Map representing the Brazilian Amazon coast between Marajó Bay and São José Bay, showing the mangrove distribution (green; source: [56]) and the economic and environmental microregions (dark gray) analyzed in the present study.

The study is located eastward of the mouth of the Amazon River, between Marajó Bay (0^o^30’ S, 48^o^ W), in the state of Pará, and São José Bay (2^o^ S, 44°15’ W), in the state of Maranhão (Fig 1). This area encompasses 68 municipalities, including the state capitals of Pará (Belém) and Maranhão (São Luís). The 11 microregions defined by the IBGE were grouped by their environmental and socioeconomic characteristics, forming a total area of 57,570 km^2^. This area includes the largest and best-preserved mangroves in Brazil [25, 54], one of the most developed mangroves in the world [30, 62]. Its landscape is derived from the combination of a small number of species and the unique local climatic and edaphic conditions [12], including 7,210 km^2^ of mangrove forest and a salt flat zone of 542 km^2^ [56]. The mangroves of this region have enormous social and economic value for the local population, providing essential resources for the production of food and the generation of income, and guaranteeing the survival of the local communities [11,63].

## Materials and methods

The present study focuses on the different types of mangrove land use already described for the Amazon coastal region [56]. Here, we considered land use as all human activities recorded within the mangrove, including the adjacent areas of salt flat (known as the “apicum” in Brazilian Portuguese), such as deforestation, roads, urban expansion, ports, salt works, aquaculture, and degradation. The term degradation herein refers to altered mangrove areas, with dead trees and few remaining individuals that still resist the dry and hypersaline soil exposed to high solar radiation [64]. We selected nine anthropogenic drivers, which are expressed by factors associated with anthropogenic features including human population density, urbanization, infrastructure, and their location in relation to the mangrove that have a direct and/or indirect influence on mangrove land use (Table 1). The spatial analyses were based on the administrative microregions defined in the database of the Brazilian Institute for Geography and Statistics, or IBGE (https://downloads.ibge.gov.br/downloads_geociencias.htm). The data were analyzed at a microregional scale, given that the municipal-level data are inadequate for a comparative analysis, in particular because many municipalities have only an incomplete dataset. All the analyses described below were run in ESRI ArcGIS 10.4.

**Table 1:** Drivers used to analyze mangrove land use due to human pressure by microregion on the Brazilian Amazon coast.

Driver	Definition	Year	Source
Population Size	Number of people	2015	GHS
Settlement	Total settlement area (km^2^)	2015	GHS
Urban Center	Number of urban center	2015	GHS
Paved Road	Total paved road length (km)	2011–2015	Present study
Unpaved Road	Total unpaved road length (km)	2011–2015	Present study
Total Road	Total road (paved + unpaved) length (km)	2011–2015	Present study
Distance from Paved Road	Average distance to the nearest paved road	2011–2015	Present study
Distance from Unpaved Road	Average distance to the nearest unpaved road	2011–2015	Present study
Distance from Urban Center	Average distance to the nearest urban center	2015	Present study

### Dataset and vectorization

**Fig 2:**
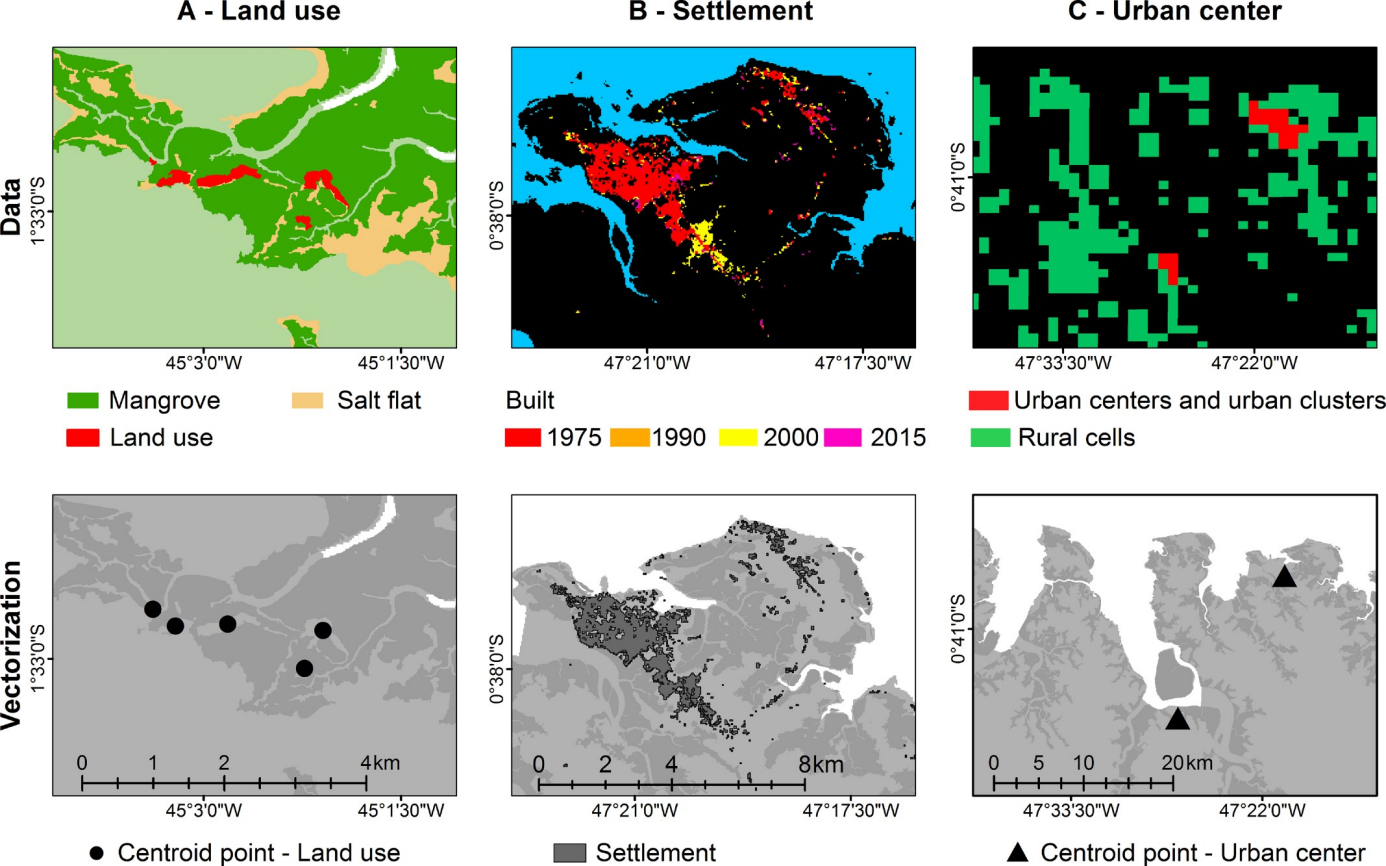
Data vectorization of the Brazilian Amazon coast. A) each record of land use is represented by a point at the centroid of a polygon of the area of mangrove affected. B) settlements recorded in 1975, 1990, 2000, and 2015 are represented by dark gray polygons. C) urban centers are represented by black triangles at the centroid of the polygon.

The patterns of use of the mangrove were diagnosed from the mapping of land use and land cover developed in 2018 from a series of RapidEye satellite images (2011–2015), using the geographic object-based image analysis approach (GEOBIA) [47]. The data refer to the type of land use recorded within the mangrove forest and adjacent salt flats. The data were retrieved in vectorial format (shapefile), with a spatial resolution of 5 m. Each record of land use was represented by a point at the centroid of a polygon of the area of mangrove affected, with the number of points being computed for each microregion (Fig 2).

The data on population density, settlements, and urban centers were obtained from the Global Human Settlement (GHS) database (https://ghsl.jrc.ec.europa.eu), developed by the Combined Research Center of the European Commission, which has collected a global temporal series of Landsat images for the period between 1975 and 2015, with elevation data being obtained from the ASTER Global Digital Elevation Map and the Shuttle Radar Topographic Mission, SRTM [65]. The GHS Built-up product was selected for the extraction of the constructed area layer, GHS-POP was selected for population density, and GHS-SMOD for the settlement model (Fig 2). In the latter case, the “urban cluster” and “urban center” classes were selected from the degree of urbanization model (GHS-SMOD). The data were first vectorized, and then the number of inhabitants, the area of the settlements, and the number of urban centers were quantified for each microregion of the Brazilian Amazon coast. The roads (paved, unpaved, and total) were mapped and classified using RapidEye satellite images obtained from the Brazilian Ministry of the Environment [66]. The images analyzed were orthorectified 3A products in the UTM projection with spatial resolution of 5 m and five spectral bands: Blue (440–510 nm), Green (520–590 nm), Red (630–685 nm), Red Edge (690–730 nm) and Near Infrared (760–850 nm) [57]. A total of 140 scenes were necessary to map the roads of the study region (S1 Table). For each scene, an image was selected that had been taken between 2011 and 2015, based on the following criteria: i) the most recent year available, ii) the lowest cloud cover, and iii) the best visual and spectral quality. The final composition of the images with their respective acquisition years is shown in S1 Table.

The roads were identified and mapped at a scale of 1:20,000, based on the vectorization of the visual interpretation of bands 2 and 3 of the RapidEye images. These bands best represent the contrast between the exposed ground of roads and adjacent features. The roads were classified as either (i) paved or (ii) unpaved. The total extension of the road network was obtained by adding the total length of the paved and unpaved roads. The classification was cross-checked by determining which digitalized roads coincided with the official map of paved roads of the Brazilian National Department of Infrastructure and Transports, DNIT (Fig 2). All other roads that did not coincide with the DNIT map were classified as unpaved. The road network of each microregion region was measured by summing the length of all the roads, in kilometers.

**Fig 3:**
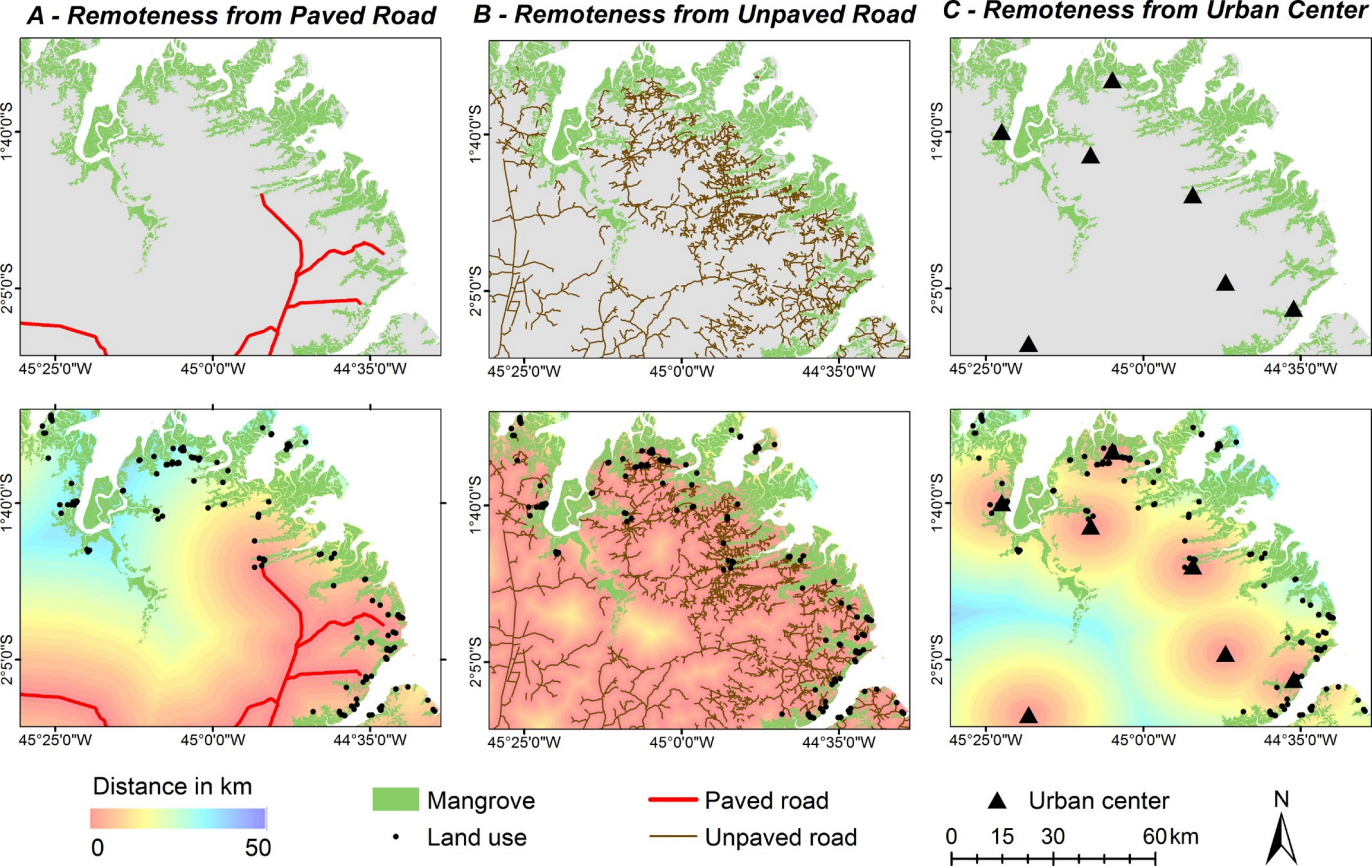
Distance (km) from the occurrences of land use in relation to A) paved roads (red lines), B) unpaved roads (brown lines), and C) urban centers on the Brazilian Amazon coast (black triangles).

The distribution of the mangrove land use in relation to the distance from paved and unpaved roads was determined by creating a buffer around each road, in parallel bands, 1 km wide, until all the occurrences of mangrove land use were included (Fig 3). The number of occurrences of mangrove land use within each buffer and the distance of each occurrence from the road were then computed for each microregion. This procedure was repeated for the urban centers.

### Data analysis

A Principal Components Analysis (PCA) was used to reduce the quantity of predictor variables (anthropogenic drivers), in order to avoid multicollinearity in regression analysis (simple and multiple) and to generate the best models to explain the relationship between the occurrence of mangrove land use (the dependent variable) and the predictor variables. As the raw data did not satisfy the assumptions of normality (Shapiro-Wilk test) and homoscedasticity (Levene test), they were log-transformed to run regression models. The variable with the greatest predictive power was determined based on the highest beta (β) value and the Pearson correlation coefficient (r). All statistical analyses were run in the BioEstat 5.0 package [67].

## Results

**Fig 4:**
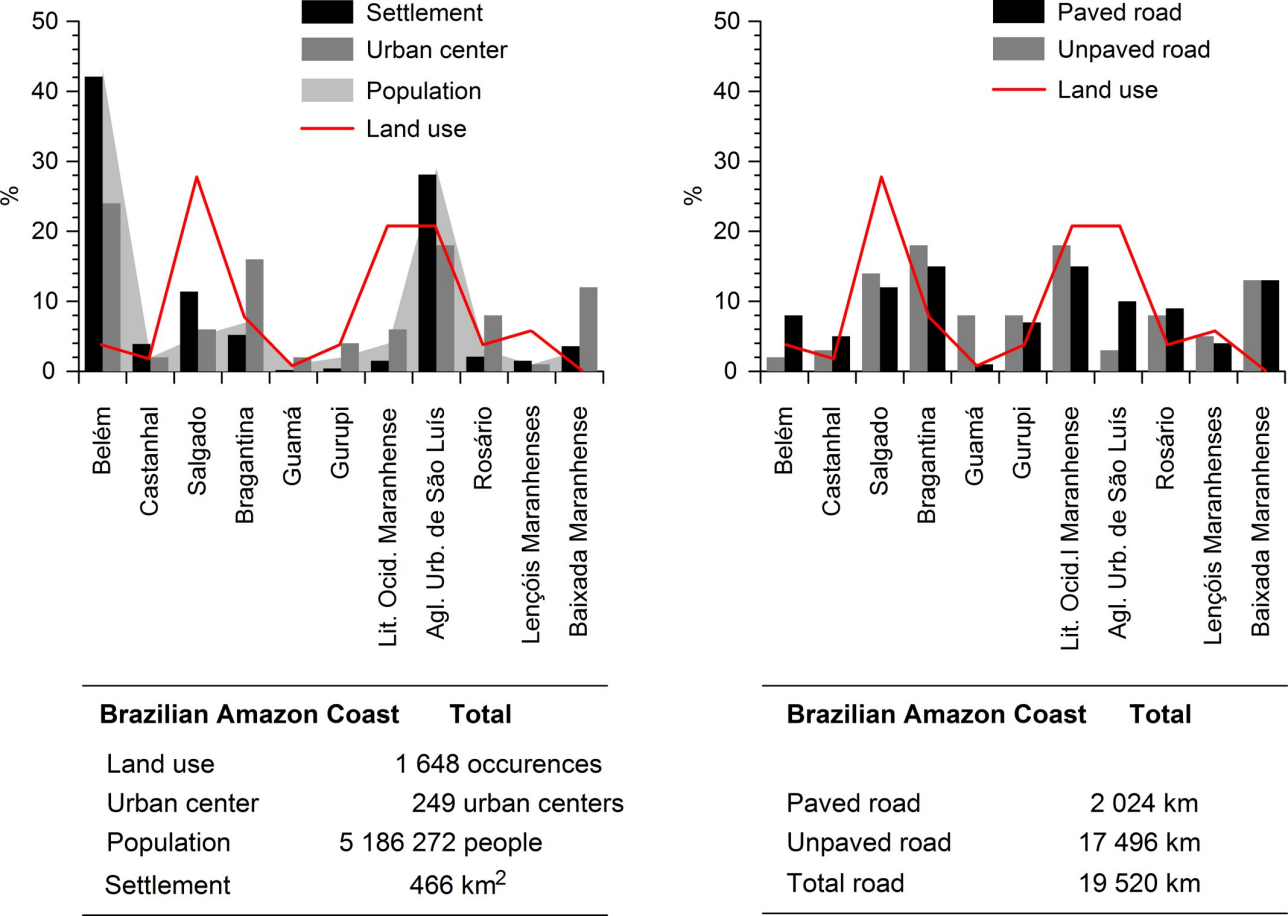
Distribution of mangrove land use and anthropogenic drivers in the different microregions of the Brazilian Amazon coast. The microregions on the x-axis are arranged in latitudinal order, i.e. from west to east (see Fig 1).

Within the study area, on the eastern Amazon coast, a total of 1648 occurrences (Fig 4,S2 Table) of mangrove land use, occupying a total area of 67.11 km^2^ [56] were recorded. This is approximately 1% of the study area. The use of mangrove habitats is concentrated primarily in the Salgado microregion, in Pará, and the Western Maranhão Coast and São Luís Urban Agglomeration microregions, in Maranhão (Fig 4). Together, these three microregions accounted for 70% of the records of anthropogenic impact in the mangrove ecosystem. By contrast, the Guamá microregion was the least used, with only 1% of the occurrences, followed by the Castanhal (2%) and Belém Metropolitan microregions, all located in Pará (Fig 4). However, the latter two microregions had only very small patches of use, which may have made them less discernible. Only the Baixada Maranhense microregion lacked anthropogenic impacts altogether, and had a well-preserved tract of mangrove.

**Fig 5:**
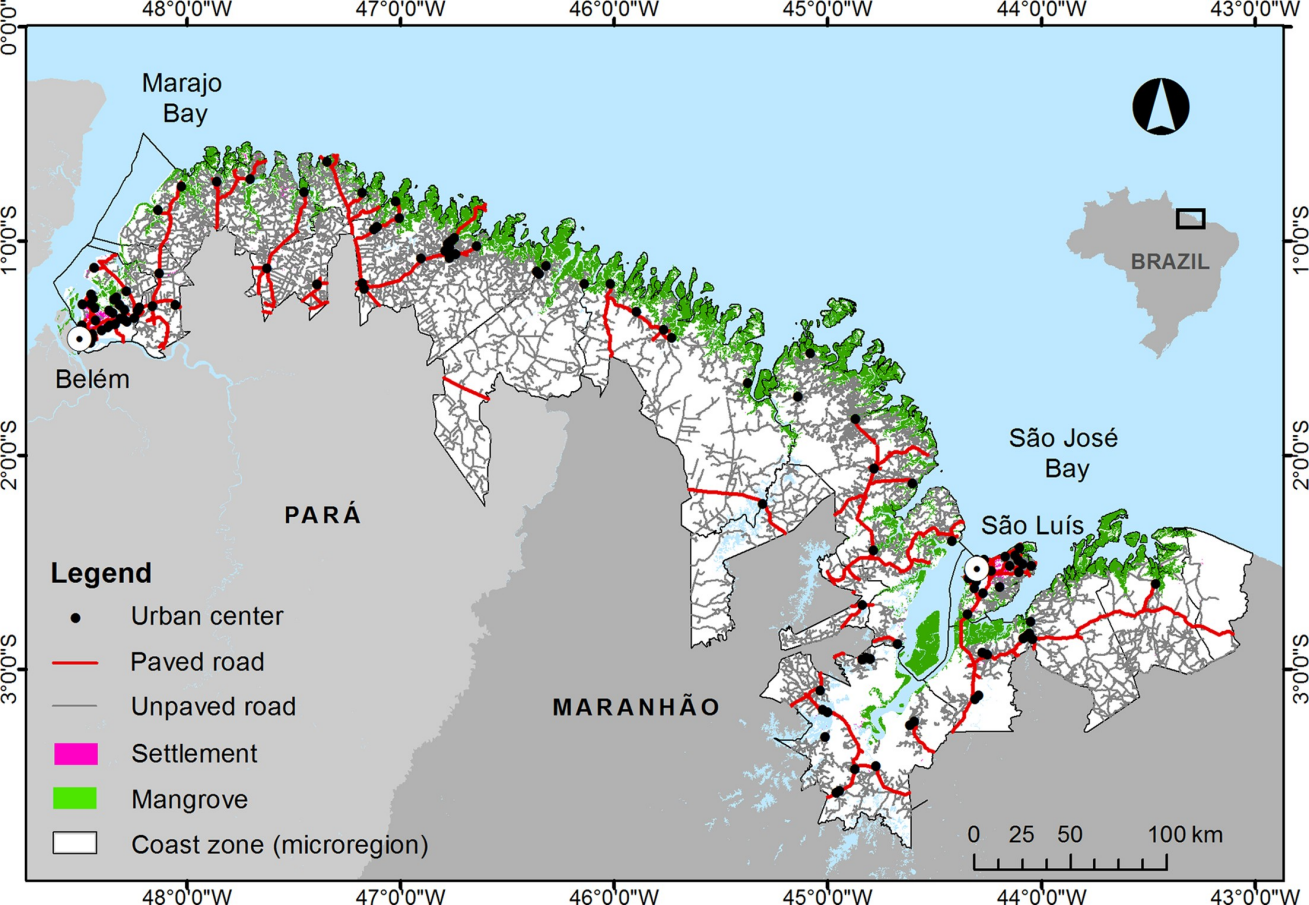
Spatial distribution of the principal anthropogenic drivers (urban centers, paved and unpaved roads, and settlements) associated with mangrove land use on the Brazilian Amazon coast.

The population of the Brazilian Amazon coast is concentrated primarily in the Belém and São Luís Urban Agglomeration microregions, which have the largest numbers of urban centers and areas of settlement (Figs 4 and 5). A number of other microregions, such as Bragantina and Salgado, are also highly urbanized, with 16% of the total number of urban centers and 11.4% of the total area of settlement, respectively. In the Salgado microregion, the municipality of Salinópolis is something of an anomaly, with high levels of real estate speculation, driven by the local tourism industry. In general, most of the Brazilian Amazon coast is sparsely populated, with vast areas still completely unoccupied.

**Fig 6:**
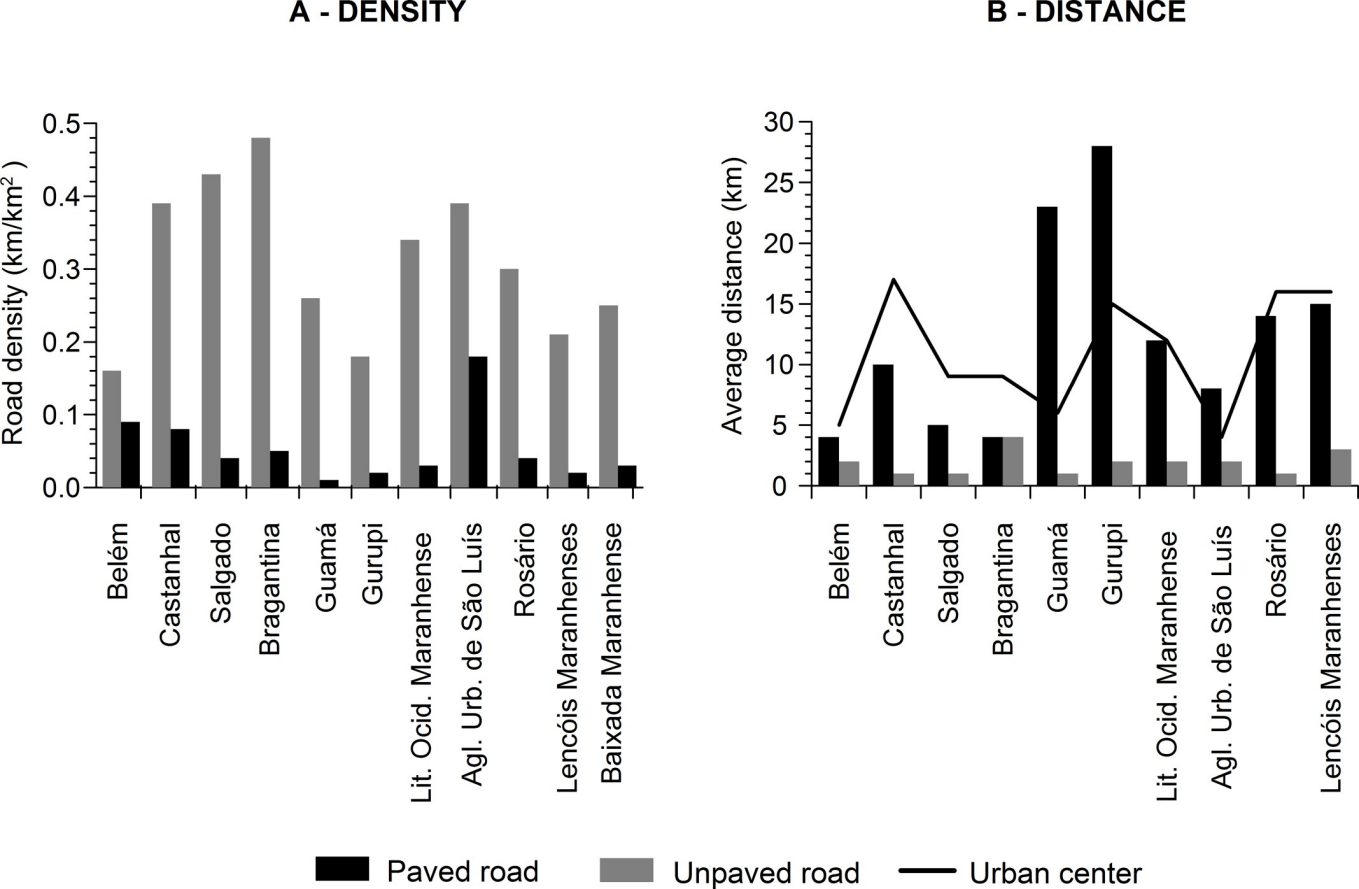
Distribution of roads in the microregions of the Brazilian Amazon coast. A) Density (km/km^2^) of paved (in black) and unpaved (in gray) roads. B) Mean distance (km) of the occurrence of mangrove land use to paved roads (in black), unpaved roads (in gray), and urban centers (black lines). The microregions on the x-axis are arranged in latitudinal order, i.e. from west to east (see Fig 1).

**Fig 7:**
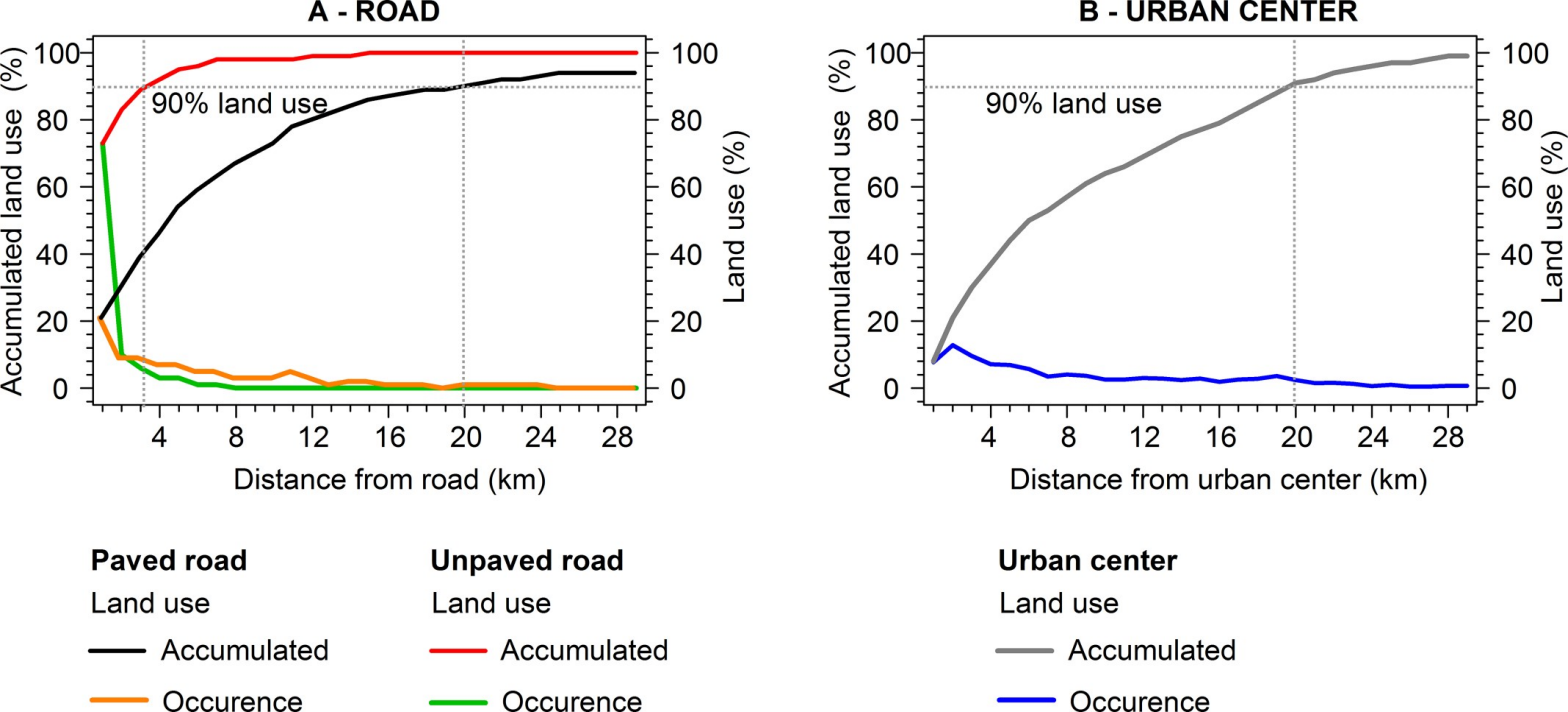
Accumulation of mangrove land use in relation to the distance from anthropogenic drivers. A) Accumulated mangrove land use (left axis) in relation to paved (black lines) and unpaved (red lines) roads. Percentage mangrove land use (right axis) in relation to paved (orange lines) and unpaved (green lines) roads. B) Accumulated mangrove land use (left axis) in relation to urban centers (gray lines). Percentage mangrove land use (right axis) in relation to urban centers (blue lines). The gray dashed line indicates the distance within which 90% of mangrove land use was registered in relation to paved roads and urban centers (20 km), and unpaved roads (3 km).

The road network of the study area has a total length of 19,520 km, most (90%) of which is unpaved (Fig 4), and extends throughout the whole of the region, as far as the edge of the mangrove (Fig 5). The other 10% of the roads (paved) are constituted primarily of federal highways. The road network has a highly variable distribution, with some microregions containing a disproportionately small percentage of the total, such as the Guamá microregion, with 7%, and Lençóis Maranhenses with only 5%, whereas the Bragantina and Western Maranhão Coast microregions had the highest percentages, both with 18%. However, the highest density (0.57 km/km^2^) was recorded in the São Luís microregion, due primarily to the contribution of paved roads, which accounted for 0.18 km/km^2^ (Fig 6A, S3 Table). The Bragantina microregion also had a relatively high density of roads, but in this case, due to the number of unpaved roads (Fig 6A). In terms of accessibility, 90% of the records of mangrove land use were concentrated within a radius of 20 km of a paved road and urban center (Fig 7, S4 Table). On the other hand, unpaved roads provided greater accessibility, with records of land use being concentrated with a radius of 3 km of this component of the landscape. A similar pattern of proximity to unpaved roads was recorded in all the microregions, especially in Castanhal (Pará), Salgado (Pará), Guamá (Pará), and Rosário (Maranhão) (Fig 6B, S5 Table). Overall, the rural roads are very common in the study area, and often extend as far as the edge of the mangrove.

**Fig 8:**
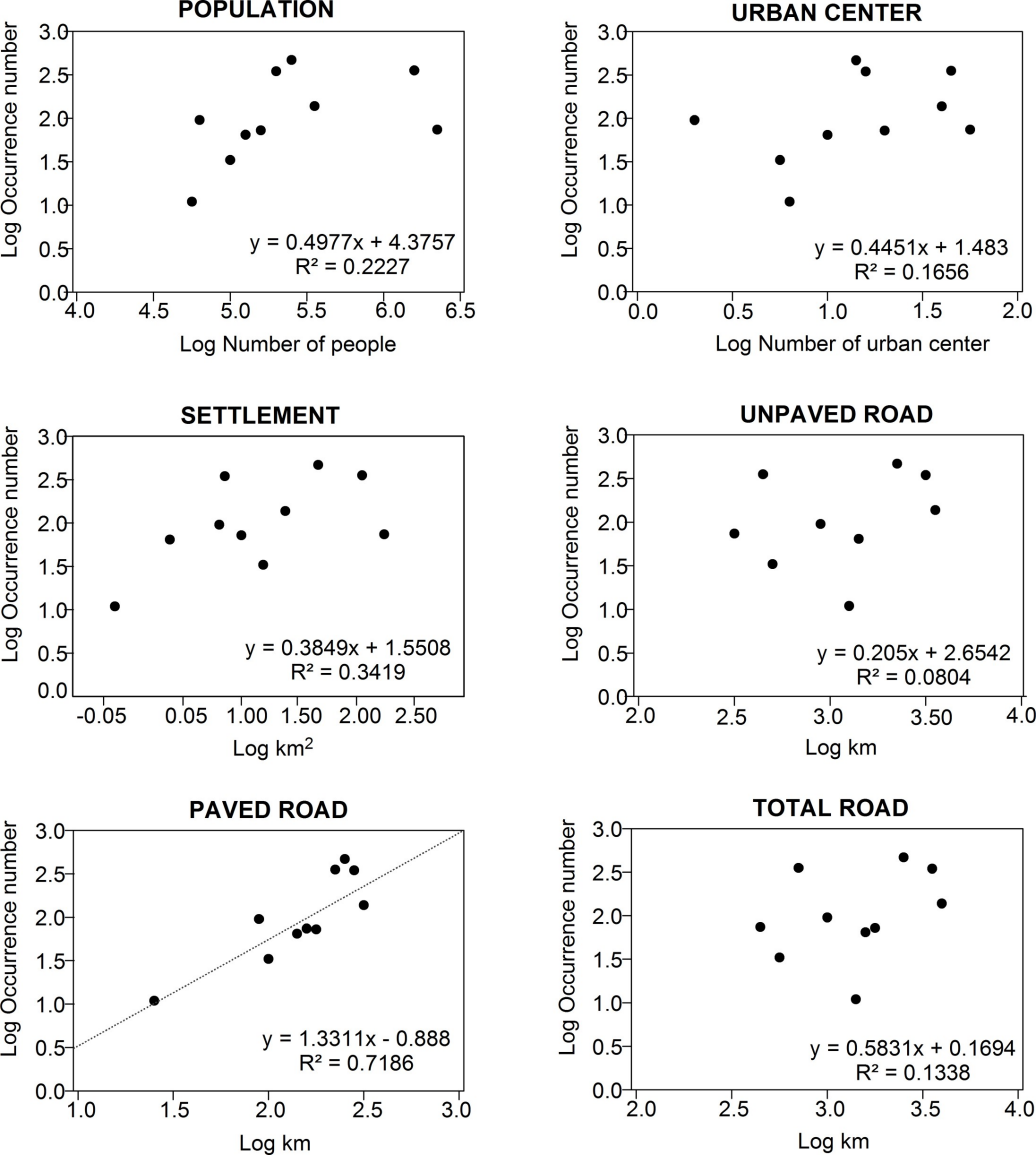
Relationship between the occurrence of mangrove land use and the distribution of anthropogenic drivers on the Brazilian Amazon coast.

The results of the PCA indicated that the first two components best explained the variation among the anthropogenic drivers (Table 2). The first component explained 45.1% of the total variance and is associated primarily with the high loadings of population density, settlement areas, and urban centers. The second component explained 34.39% of the variance, and was determined by the loadings for paved and unpaved roads, and all roads together. The drivers of both components (the first and second components) were then correlated with the rates of occurrence of anthropogenic activities in the mangrove. The simple linear regression (Table 3, Fig 8) found a significant association only between mangrove land use and paved roads (ß = 1.33; r = 0.72; F = 20.45, p < 0.05). The results of the multiple (stepwise) regression analysis also indicated significant associations for the models that included the factors population density, area of settlement, and the presence of roads (Table 3). In all the models generated, paved roads were the factor that had the greatest power for the prediction of the anthropogenic activities recorded in the Amazonian mangroves.

**Table 2:** Results of the principal components analysis (PCA) of the anthropogenic drivers.

Driver	CP_1_	CP_2_	CP_3_
**Population**	0.4803	-0.0964	0.0397
**Settlement**	0.4803	-0.1043	-0.0591
**Urban center—quantity**	0.4641	0.1204	0.1016
**Unpaved road**	-0.1629	0.52	-0.1818
**Paved road**	0.1506	0.5054	-0.1146
**Total road**	-0.1416	0.5308	-0.1804
**Distance from paved road**	-0.3297	-0.2382	0.1387
**Distance from unpaved road**	0.0934	0.3148	0.8903
**Distance from urban center**	-0.3694	-0.0442	0.3065
**Eigenvalues**	4.0596	3.0949	0.7882
**% Total Variance**	45.11	34.39	8.76
**% Accumulated**	45.11	79.49	88.25

CP_1…_CP_3_ = First…Third Principal Component.

**Table 3:** Results of the simple linear and multiple (stepwise) regressions between the occurrence of land use in the mangrove and anthropogenic drivers on the Brazilian Amazon coast. p = significance level (α = 0.05); a-Population; b-Settlements; c-Urban centers; d-Unpaved road; e-Paved road; f-Total roads.

Variable	r (Pearson)	β	R^2^	F	QM Erro	p
**Simple Linear Regression**						
**Population**	0.4719	0.4474	0.2227	2.2917	0.2207	0.1665
**Settlement**	0.5847	0.3849	0.3419	4.1563	0.1868	0.0737
**Urban center**	0.4069	0.4451	0.1656	1.5872	0.2369	0.2423
**Unpaved road**	0.2836	0.3923	0.0804	0.6996	0.2611	0.5684
**Paved road**	0.8477	1.3311	0.7188	20.4319	0.0798	0.0023
**Total road**	0.3657	0.5831	0.1338	1.2353	0.2459	0.2991
**Multiple Linear Regression (Stepwise)**						
**a;b;c;d;e;f**	0.954	-	0.9101	5.0613	0.0681	0.1060
**a;b;c;e;f**	0.9531	-	0.9084	7.9369	0.0520	0.0356
**a;b;c;e**	0.8948	-	0.8007	5.0227	0.0905	0.0541
**a;b;e**	0.8551	-	0.7312	5.4398	0.1018	0.0383
**a;e**	0.851	-	0.7241	9.1874	0.0895	0.0114
**e**	0.8478	-	0.7188	20.454	0.0798	0.0023

## Discussion

The present study provided a synoptic perspective on the different human activities that exploit areas of mangrove habitat on the Brazilian Amazon coast, and the anthropogenic factors that drive this process. This region is sparsely populated, with the exception of the Belém microregion, in Pará, and the São Luís Urban Agglomeration in Maranhão. This is reflected in the high level of conservation of the mangroves of northern Brazil, with the habitat in most microregions presenting low levels of exploitation.

While still occurring at relatively low levels, human intervention in the mangrove is clearly driven by the road network, in particular the paved roads. Other factors, such as population density, the expansion of settlements, and the quantity of urban centers, and different combinations of these drivers, had less influence on mangrove land use. Overall, then, the lack of an adequate network of paved highways throughout much of the region contributes to the low levels of anthropogenic pressure found in the mangrove of the Brazilian Amazon coast, despite the local predominance of unpaved roads, which actually provide better access to the mangrove. This scenario clearly determines the extremely low rates, of less than 1% [56], of mangrove land use on the Amazon coast.

This scenario contradicts global trends, however, given that most coastal zones are characterized by high population densities [46, 47], which drive profound impacts on their natural ecosystems [68], in particular mangroves [35]. A number of studies have indicated that population density is one of the principal drivers of human impact on the mangrove [35, 51, 52, 69]. Under the current scenario, population density is only of secondary relevance on the Brazilian Amazon coast. This region is characterized by extensive demographic voids (Fig 5), and only 8% of the population of Pará live in the coastal zone [70], whereas in other Brazilian regions, the coastal zone is inhabited by up to 40% of the population [71]. In the Amazon region, with the exception of the metropolitan areas (Belém and São Luís), the economies of the coastal microregions are based on traditional local subsistence activities, in particular, artisanal fisheries, and the exploitation of other mangrove resources [11, 32, 72]. In this scenario, paved roads are the principal drivers of mangrove land use on the Brazilian Amazon coast.

As in other areas of the Amazon basin [59–61] and the world [73], then, roads are the principal drivers of deforestation in the mangrove [28, 51, 74]. Although roads support social and economic development [75–77], they may also contribute to the degradation of natural environments [77, 78], in particular, in developing countries [52]. On the Brazilian Amazon coast, the road network is formed primarily (90%) by rural, unpaved roads, which is typical of the global scenario [79] and the rest of Brazil [80]. Despite this configuration, the better, paved roads tend to drive the spatial distribution of human impacts in the mangrove.

One clear example of this process is the PA-458 state highway, which links the town of Bragança to the Vila dos Pescadores community on the Ajuruteua Peninsula, in Pará. This 26 km-long highway was constructed in the 1970s, creating a linear corridor of disturbance that interrupts the flow of water and has caused the degradation of 6 km^2^ of Avicennia forest on the left side of the road [81]. While covering only a small area, this feature is considered to represent the principal impact on the mangrove of the Brazilian Amazon coast [64]. A more general analysis also reinforces the conclusion that paved roads have the most negative impacts on the mangrove [52]. A similar tendency has been recorded in Amazonian rainforest, where paved roads lead to more extensive degradation of the forest than unpaved roads [82].

It is important to note that road transportation is prioritized in Brazil, where many highways have been built in the coastal zone. However, the road network in the region of the Amazon coast is still underdeveloped in comparison with other regions, in particular, in southern and southeastern Brazil, where the paved road network is far more extensive [83]. The scenario observed on the Amazon coast has thus contributed to the conservation of the region’s mangroves, which are largely unscathed. The federal Transoceanic highway (BR-308), which links the municipalities of Capanema, in Pará, and Alcântara, in Maranhão, is one case of potential risk, ranging from socioeconomic development to the maintenance of the ecosystem services provided by the mangrove. Since 1999, when it came under federal jurisdiction, this highway has still yet to be finalized and paved. Some stretches in Maranhão are still isolated, and paving works between Bragança and Viseu, both in Pará, were only begun in 2016 [84]. Once concluded, this highway will be important for regional integration, by reducing the distance between its two principal metropolises, Belém and São Luís, facilitating the flow of merchandise, and contributing to the socioeconomic growth of the northern coast of Brazil [85].

An additional 636 km of road will also be constructed in areas adjacent to the mangrove, traversing the Bragantina, Guamá, Gurupi, and Western Maranhão Coast microregions, which are currently the areas with the most conserved mangrove and the lowest rates of land use in the Amazon coastal zone. One other, well-consolidated case, is the BR-101 federal highway, which runs the length of the eastern coast of Brazil, from Rio Grande do Norte, at the northeastern tip of the country, to Rio Grande do Sul, at its southernmost extreme. The construction of this highway, in the 1970s, led to alterations along the whole coastline, including the degradation of mangrove habitats [50].

The effective diagnosis of the influence of this factor on areas of mangrove habitat depends on the systematic understanding of the biases that determine the differential investment in the region’s infrastructure and the expansion of its road network. To begin with, the construction of paved highways leads to the implantation of additional roads and the expansion of the network, providing access to previously unexploited territorial frontiers [77, 86, 87]. The long-term consequences of the construction and paving of major federal highways for the degradation of natural resources in the Amazon basin have been well documented in cases such as that of the BR-010 highway, which links the Amazonian city of Belém, in Pará, to the federal capital, Brasília (Federal District), in the center of the country [76], the BR-163 highway, which links the central Amazonian city of Santarém (Pará) to Cuiabá (Mato Grosso), and the BR-364 highway, in the western Amazonian state of Rondônia [88].

The second point to be considered is the accessibility of rural roads. The mangrove areas most affected by anthropogenic impacts are located in the proximity of unpaved roads. As the unpaved road network has grown, the mangroves of the Brazilian Amazon coast have become increasingly accessible to human activities, given that 90% of the occurrences of mangrove land use were detected within a 3-km radius of these roads, a pattern similar to that found in the rainforest of the Amazon basin, where all deforestation was recorded within a 5.5 km radius of an unpaved road [73]. A similar pattern was also recorded in Thailand, for example, where forest was most impacted within a radius of 1 km from the nearest highway [76].

The third question here is that the improvement of the road network contributed to the access of major urban centers [77], by improving conditions for transportation and reducing costs, while also attracting immigrants, and facilitating the commerce in primary resources, including those extracted from the mangrove [89]. A delicate bias exists in this process, when the development of roads may result in social problems for traditional local communities. The mangrove of the Ajuruteua Peninsula, in Pará, is an important component of this process, with 80% of the natural resources extracted from this area being destined for regional markets [11]. This has a negative effect on the activities of the residents that harvest natural resources from local estuaries, by reducing the value of their labor in the productive chain, as well as transforming sustainable practices into the predatory exploitation of natural resources. In this way, the expansion of the road network triggers a sequence of events through which the facilitation of access to the mangrove contributes to the advance of anthropogenic impacts that, in turn, lead to fundamental socioeconomic changes and major ruptures in the ecological functions, and the goods and services provided by this ecosystem.

However, it is important to note that the factors highlighted in the present study are not the only processes that influence the loss of areas of mangrove on the Brazilian Amazon coast. Anthropogenic modifications are driven by a combination of factors, including biophysical, social, economic, and political processes [76]. For example, land use in the mangrove may be influenced by the model of regional economic development, that is, the larger the GDP, the greater the loss of mangrove habitat [27], although this same study also showed that the larger the number of protected areas, the greater the area of mangrove forest. In some regions, in fact, protected areas are a driving force that contribute to the conservation of the mangrove, as in the case of the Sundarbans, which straddle the India–Bangladesh border, Phang Nga in Thailand, and Matang in Malaysia [29]. This same effect is observed in other systems, such as the Amazonian rainforest, where protected areas have mitigated the negative impacts of anthropogenic processes [73], including a reduction in the rate of road building [61]. The consolidation of the system of protected areas is a promising strategy for the Brazilian Amazon coast, which already has 16 conservation units, created to satisfy the social, economic, and environmental needs of the region’s traditional estuarine-coastal communities, as well as regulating and protecting the natural resources of the coastal zone [32]. It is also important to consider the influence of bodies of water (e.g., rivers, channels, and creeks), on the exploitation of mangrove resources. The accessibility of the mangrove is enhanced considerably by these watercourses, which should be considered to be a key factor in coastal systems, by facilitating human intervention, as observed in the Amazon rainforest, where deforestation typically occurs within a 1-km radius of navigable waterways [73].

Overall, then, the present analysis of the current scenario of the world’s largest continuous tract of mangrove forest indicted that the expansion of the road network of the Brazilian Amazon coast, while being considered a byword for economic development, is the primary predictor of anthropogenic impact through mangrove land use. Paved roads support the expansion of the unpaved road network, which facilitate access to the mangrove ecosystem, even though the region is still sparsely-populated. Given the present scenario, preventive measures should be considered to be a priority, together with incentives for the more systematic application of the legislation that protects the mangrove. In Brazil, all mangrove is considered an Area of Permanent Preservation (APP), regulated by the Brazilian Forest Code (Federal Law 12651/2012), which determines the parameters for the exploitation of areas of native vegetation. It is also essential that the conservation units that have been established within the coastal zone are protected more effectively, to allow them to offset anthropogenic pressures through participative management strategies that involve the traditional local communities. Ultimately, master plans for local, regional, and national development must take the ecological features of the mangrove into consideration, in order to avoid impacting irrevocably the stability and development of this ecosystem. All these findings reinforce the need for an effective system of environmental governance for the coastal zone based on ecologically-sound economic and development policies, in order to guarantee the long-term conservation and sustainability of the Brazilian Amazon coast, and one of the world’s most important mangrove systems.

## Supporting information

S1 Table Scenes and dates of the RapidEye images used to map the roads on the Brazilian Amazon coast. The RapidEye satellite images were obtained from the Brazilian Ministry of the Environment [55].
(PDF)

S2 Table Dataset of mangrove land use and anthropogenic drivers in the different microregions of the Brazilian Amazon coast. (XLSX)

S3 Table Density of the roads (unpaved, paved, and total) and the populations of the different microregions of the Brazilian Amazon coast. (XLSX)

S4 Table Land use in relation to the distance (km) from paved and unpaved roads and urban centers. (XLSX)

S5 Table Average distance (km) of use in mangroves in relation to the roads (paved and unpaved) and urban centers in the micro regions of the Brazilian Amazon coast. (XLSX)

S1 File Shapefiles of mangrove land use and roads on the Brazilian Amazon coast. (RAR)
