# Supplementary material for: The effect of anthropogenic drivers on spatial patterns of mangrove land use on the Amazon coast
Source: PLoS One. 2019 Jun 26;14(6):e0217754. doi: 10.1371/journal.pone.0217754 (PMC6594664; doi:10.1371/journal.pone.0217754)
Supplement: S1 Table — The RapidEye satellite images were obtained from the Brazilian Ministry of the Environment [55]. (PDF) [file pone.0217754.s001.pdf]

S1 Table. Scenes and dates of the RapidEye images used to map the roads on the Brazilian Amazon coast. The RapidEye satellite images were obtained from the Brazilian Ministry of the Environment [55].

N	Tile	Data		N	Tile	Data
01	2338801	2012-08-03		47	2338306	2015-07-15
02	2338802	2014-11-25		48	2338307	2013-09-18
03	2338803	2014-08-01/2015-06-27		49	2338308	2015-09-20
04	2338804	2015-06-23		50	2338309	composition
05	2338701	2012-11-16/2015-08-22		51	2338310	2012-09-16
06	2338702	2014-11-23		52	2338311	composition
07	2338703	2012-09-13/2014-11-23		53	2338312	2013-01-04
08	2338704	2015-06-23		54	2338313	2011-06-05
09	2338705	2013-12-24		55	2338314	2011-06-05
10	2338706	2014-12-12/2014-11-18		56	2338315	2013-12-11
11	2338707	2013-09-09/2015-11-29		57	2338316	2011-06-06
12	2338708	2012-10-19		58	2338206	2015-09-12
13	2338601	2012-08-02		59	2338207	2015-09-12
14	2338602	2012-09-13		60	2338211	2015-11-15
15	2338603	2014-11-01		61	2338212	composition
17	2338605	2015-08-2015		62	2338213	2011/06/05
18	2338606	2014-10-29		63	2338214	2011/06/05
19	2338607	2012-07-31		64	2338215	2013/12/11
20	2338608	2014-11-18		65	2338216	2015-10-23
21	2338609	22013-07-31		66	2338217	2015-01-29
22	2338610	2012-09-26		67	2338106	2012-10-24
23	2338611	2011-11-17		68	2338107	2015-09-12
24	2338502	2012/09/13		69	2338112	2015-11-15
25	2338503	2012-09-13		70	2338113	2015-07-18
26	2338504	2012-09-13		71	2338114	2014-09-27
27	2338505	2011-07-28		72	2338115	2013-12-11
28	2338506	2012-07-31		73	2338116	2013-12-24
29	2338507	2015-07-15		74	2338117	2014-05-03
30	2338508	2014-12-11		75	2338012	2015-07-18
31	2338509	2014-12-11		76	2338013	2014-11-06
32	2338510	2014-12-10/2014/12/27		77	2338014	2014-09-27
33	2338511	2014-12-17		78	2338015	2012-10-31
34	2338512	2014-11-14		79	2338016	2011-10-15
35	2338515	2015-07-18		80	2338017	2015-07-26
36	2338406	2012-07-31		81	2338020	2011-10-27
37	2338407	2012-07-31		82	2338021	2011-10-07
38	2338408	2015-11-26		83	2338022	2015-08-08
39	2338409	2015-10-03		84	2337912	composition
40	2338410	composition		85	2337913	2015-07-18
41	2338411	2013-07-26		86	2337915	composition
42	2330412	2015-10-17		87	2337916	2012-10-06
43	2330413	2011-06-05		88	2337917	2012-10-06
44	2338414	2013-11-02		89	2337918	2015-07-07
45	2338415	2013-03-09		90	2337919	2013-08-30
46	2338416	2011-10-15		91	2337920	2015-07-22

N	Tile	Data		N	Tile	Data
92	2337921	2013-10-22		114	2337614	2014-09-27
93	2337922	2015-08-08		115	2337615	2013-10-05
94	2337923	2013-05-31		117	2337616	2012-06-08
95	2337812	2014-11-06		118	2337617	2012-06-08
96	2337815	composition		119	2337620	2015-08-09
97	2337816	2012-11-02		120	2337621	2014-10-31
98	2337817	2015-08-07		121	2337514	2014-10-25
99	2337818	2015-09-10		122	2337515	2013-10-05
100	2337819	2015-09-10		123	2337516	2012-11-02
101	2337820	2013-07-16		124	2337414	2014-09-27
102	2337821	2015-08-08		125	2337415	2014-09-27/2014-11-27
103	2337822	31/10/2014		126	2337416	2014-11-27
104	2337823	2015-10-07		127	2238727	2014-08-17
105	2337714	2015-08-02		128	2238728	2014-08-17
106	2337715	2015-10-25		129	2238626	2014-08-18
107	2337716	2015-08-07		130	2238627	2012-08-02
108	2337717	2015-08-07		131	2238628	2015-08-22
109	2337718	2015-08-07		132	2238526	2013-08-01
110	2337720	2014-10-13		133	2238527	2013-08-01
111	2337721	2015-08-08		134	2228528	2015-08-22
112	2337722	2014-10-13		135	2238426	2015-06-28
113	2337723	2015-07-15		136	2238427	2013-08-01
